# Automatized detection of uniparental disomies in a large cohort

**DOI:** 10.1007/s00439-024-02687-w

**Published:** 2024-07-16

**Authors:** Johanna Moch, Maximilian Radtke, Thomas Liehr, Thomas Eggermann, Christian Gilissen, Rolph Pfundt, Galuh Astuti, Julia Hentschel, Isabell Schumann

**Affiliations:** 1 Institute of Human Genetics, Leipzig University, Leipzig, Germany; 2 Institute of Human Genetics, Jena University, Jena, Germany; 3 Institute of Human Genetics and Genomic Medicine, Aachen University, Aachen, Germany; 4 Department of Human Genetics, Radboud University Medical Center, Nijmegen, the Netherlands

**Keywords:** Uniparental disomy, Heterodisomy, NGS, Regions of homozygosity, ROH, Inheritance ratio, Web app, AltAFplotter

## Abstract

Uniparental disomy (UPD) is the inheritance of both homologues of a chromosome from only one parent. The detection of UPDs in sequencing data is not well established and a common gap in genetic diagnostics. We applied our in-house UPD detection pipeline to evaluate a cohort of 9212 samples, including multigene panels as well as exome sequencing data in a single, duo or trio constellation. We used the results to inform the design of our publicly available web app altAFplotter. UPDs categorized as heterodisomy, whole chromosome or segmental isodisomy were identified and validated with microsatellites, multiplex ligation-dependent probe amplification as well as Sanger sequencing. We detected 14 previously undiagnosed UPDs including nine isodisomies, four segmental isodisomies as well as one heterodisomy on chromosome 22. We characterized eight findings as potentially causative through homozygous pathogenic variants or imprinting disorders. Overall, our study demonstrates the utility of our UPD detection pipeline with our web app, altAFplotter, to reliably identify UPDs. This not only increases the diagnostic yield of cases with growth and metabolic disturbances, as well as developmental delay, but also enhances the understanding of UPDs that may be relevant for recurrence risks and genetic counseling.

**Supplementary Information:**

The online version contains supplementary material available at 10.1007/s00439-024-02687-w.

## Introduction

Uniparental disomy (UPD) describes the origin of both homologues of a chromosome from the mother (UPD(mat)) or the father (UPD(pat)) (Engel and DeLozier-Blanchet 1991). They can divided in two classes: (1) if both homologues are two copies of a single parental homologue it is termed isodisomy (iUPD) and (2) if both homologues originate from a single parent and reflect both parental homologues it is termed heterodisomy (hUPD) (Engel and DeLozier-Blanchet 1991). Whereas complete hUPDs typically result from a nondisjunction error in meiosis I; complete iUPDs arise through mitotic or nondisjunction error in meiosis II (Benn 2021). The main mechanisms forming UPDs are trisomic or monosomic rescue, gamete complementation or postfertilization error (Robinson 2000). Segmental UPDs covering only parts of the affected chromosome can arise through mitotic recombination between the parent’s homologous chromosomes before one extra chromosome is removed during a trisomic rescue (Eggermann et al. 2018). Additionally, segmental UPDs which include iUPDs and hUPDs on the same chromosome due to meiotic recombination are referred to as mixed UPDs (Kotzot 2001).

UPDs are not necessarily pathogenic and only result in clinical consequences when chromosomal disturbances, imprinting effects or homozygosity of autosomal recessive variants occur (Eggermann et al. 2018). Furthermore, some chromosomes are more likely to be clinically relevant than others if UPDs occur, due to known imprinting disorders (Kotzot and Utermann 2005) involving chromosome 6, 7, 11, 14, 15 and 20 (Gaudio et al. 2020). UPDs can be associated with small supernumerary marker chromosomes (Kotzot 2002) or other chromosomal aberrations such as mosaic aneuploidy which can be the result of the mechanism behind UPD formation, like incomplete trisomic rescue, and could contribute to phenotypic abnormality (Liehr 2010). This makes UPD diagnostics even more important and can have an effect on genetic counseling even if the UPDs are not pathogenic themselves (Chien et al. 2022).

So far, the UPD frequency is not definitively known. In literature, the UPD occurrence varies from 0.05 to 0.6% (Nakka et al. 2019). Established molecular methods to detect UPDs include single-nucleotide polymorphism-based (SNP) microarrays detecting isodisomies through long contiguous stretches of homozygosity (Papenhausen et al. 2011). Methods for targeted UPD testing in case of a phenotype suggestive of a disorder caused by UPD can include multiplex ligation-dependent probe amplification using methylation specific sites or short tandem repeat analysis (Eggermann 2020). There have also been different approaches to identify UPDs in exome sequencing (ES) data since next-generation sequencing (NGS) methods have become the first line technique in routine genetic diagnostics (Tran Mau-Them et al. 2021).

There are several algorithms available for analyzing UPDs out of NGS data (Bis et al. 2017; Yauy et al. 2020; Scuffins et al. 2021; Wang et al. 2021). All studies used regions of homozygosity (ROH) to detect isodisomies. In contrast, the detection of heterodisomies remains challenging, although various approaches have been proposed. For example, Yauy and colleagues used ES data in a trio cohort to trace back the parental origins of each SNP (Yauy et al. 2020). Scuffins and colleagues on the other hand categorized parental origins through Mendelian inheritance errors (Scuffins et al. 2021). Due to the wide variety of approaches, we wanted to streamline UPD detection and evaluation using a newly designed web app called altAFplotter, which utilizes ROHs as well as inheritance ratios to bridge the diagnostic gap (Radtke et al. 2023).

A lot of knowledge about UPD analysis has been generated based on molecular methods such as microarray data (Papenhausen et al. 2011; Hoppman et al. 2018). However, new standards need to be established using ES data. Therefore, more studies and experience using NGS data as input in UPD diagnostics, are needed. This study demonstrates the application of our UPD detection pipeline in combination with the altAFplotter on our cohort consisting of 9212 cases in a single, duo or trio constellation sequenced with several enrichment kits for multigene panel and ES. We have determined the prevalence of UPD cases within our cohort and propose the use of whole methylome sequencing to analyse imprinting defects of newly identified UPDs. Furthermore, the study leads to the implementation of the altAFplotter into our routine diagnostic pipeline and highlights the utility of the one-in-all approach of ES in genetic diagnostics.

**Fig. 1:**
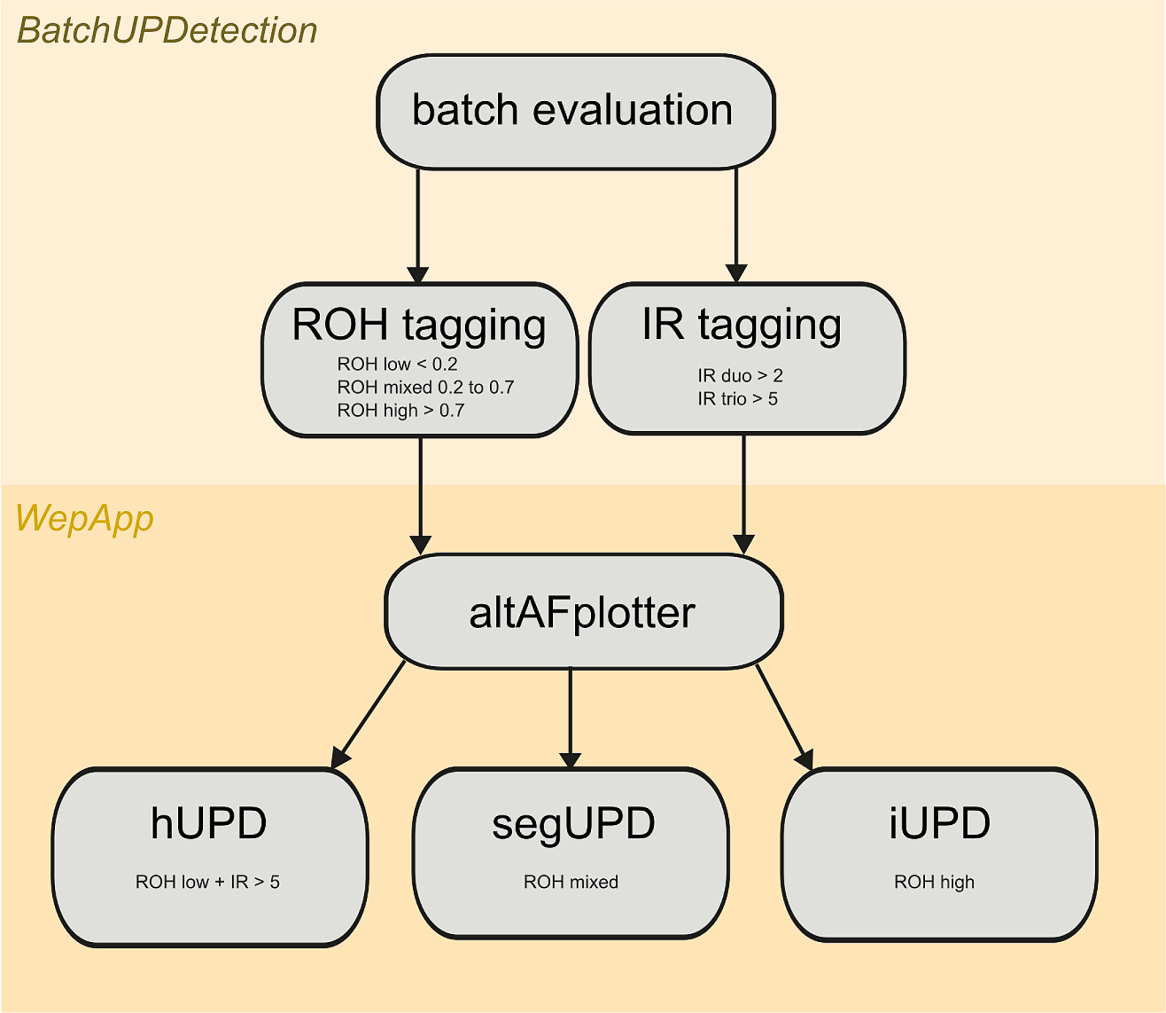
Methodological overview of UPD analysis in the large cohort. Batch evaluation of the entire cohort was conducted using the *in-house* pipeline BatchUPDetection. ROHs and IRs were tagged using defined cut offs (ROH low < 0.2; ROH mixed 0.2–0.7; ROH high > 0.7; IR duo > 2; IR trio > 5). Conspicuous samples were then verified manually with the web app altAFplotter. Note that cut offs were adjusted to the family set-up: trio, duo and single analysis. Abbreviations: hUPD; heterodisomy, IR; inheritance ratio, iUPD; isodisomy, ROH; region of homozygosity, seqUPD; segmental UPD, UPD; uniparental disomy

## Materials and methods

### Cohort composition and genetic analyses

**Fig. 2:**
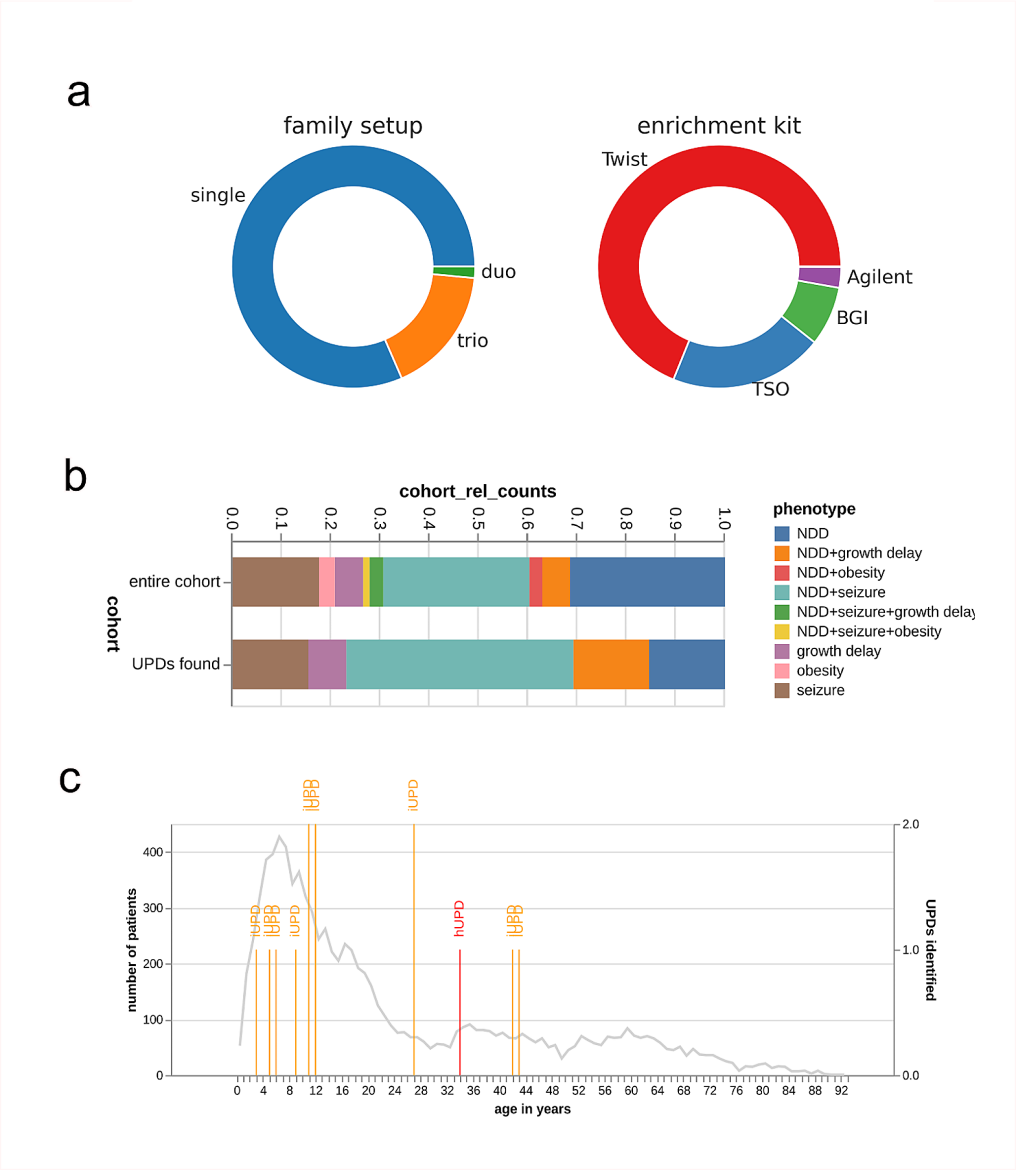
Cohort composition. **(a)** Left diagram represents the family set-up in the cohort; note that most of the analyses are single (affected patient only) examinations. Right diagram indicates enrichment kits used for genetic diagnostics in the large cohort; note that also multigene panel analyses were included (TruSightOne, TSO). **(b)** Phenotype composition of the cohort (top) compared to the phenotype composition of the patients, where UPDs have been identified (bottom) **(c)** Age distribution of the cohort (grey line, left axis) and number of UPDs identified at the respective age (iUPDs in orange, hUPDs in red, right axis). Note that most of the individuals are in the age range of 0–20 years. Abbreviations: hUPD; heterodisomy; iUPD; isodisomy, NDD; neurodevelopmental delay

Our cohort comprised 9212 individuals with mainly rare diseases including 1557 parent-child-trios, 146 duos and 6853 single NGS analyses. We excluded 656 samples due to suspected consanguinity (Fig. 2, Supplementary Figure S1, S2). The data was collected as part of our routine genetic diagnostics at the Institute of Human Genetics, Leipzig University from 2019 to 2023. Enrichment and library preparation were performed using TWIST Human Core Exome Kit (TWIST Bioscience, San Francisco, CA, USA), SureSelect Human All Exon V7 (Agilent Technologies, Santa Clara, CA, USA), BGI Exome Capture 59 M Kit (BGI, Shenzhen, China) and TruSightOne multigene panel (4813 genes, Illumina^®^, San Diego, CA, USA).

### UPD detection in NGS data

For UPD detection we used our in-house UPD detection pipeline (https://github.com/HUGLeipzig/BatchUPDetection) and manual curation via the web app altAFplotter: altafplotter.uni-leipzig.de (Radtke et al. 2023) based on identifying runs of homozygosity (ROH) as well as inheritance ratios (IR). ROHs indicate a potential isodisomy; IR indicates the inheriting parent as well as a potential heterodisomy (only applicable for duo and trio setups, Fig. 1). Positive controls and known UPDs were used to determine cutoffs to reliably detect isodisomies and heterodisomies: chromosomes covered by > 70% ROH were rated as “high” and indicate a potential iUPD, covered by 20–70% as “high mixed” and indicate a potential segmental iUPD and < 20% as “low” and indicate that they are not conspicuous for iUPD. For trio and duo UPD analysis we combined the parameters ROH and IR. Hereby, the limit values for ROH are similar to a single analysis. An inheritance ratio > 2 in duos and > 5 in trios was marked as “high” and suggestive of heterodisomy. The batchUPDetection workflow outputs a series of graphs to allow batch evaluation of these parameters for cohorts of any size and a list of all chromosomes of all samples with corresponding tags, which can be easily filtered to identify chromosomes with possible UPDs.

### UPD Validation

UPD validation was performed with microsatellites, Sanger sequencing and multiplex ligation-dependent probe amplification as previously described (Moch et al. 2023). Identified UPDs were reported back to the affected individuals but no clinical validation was conducted so far.

## Results

**Fig. 3:**
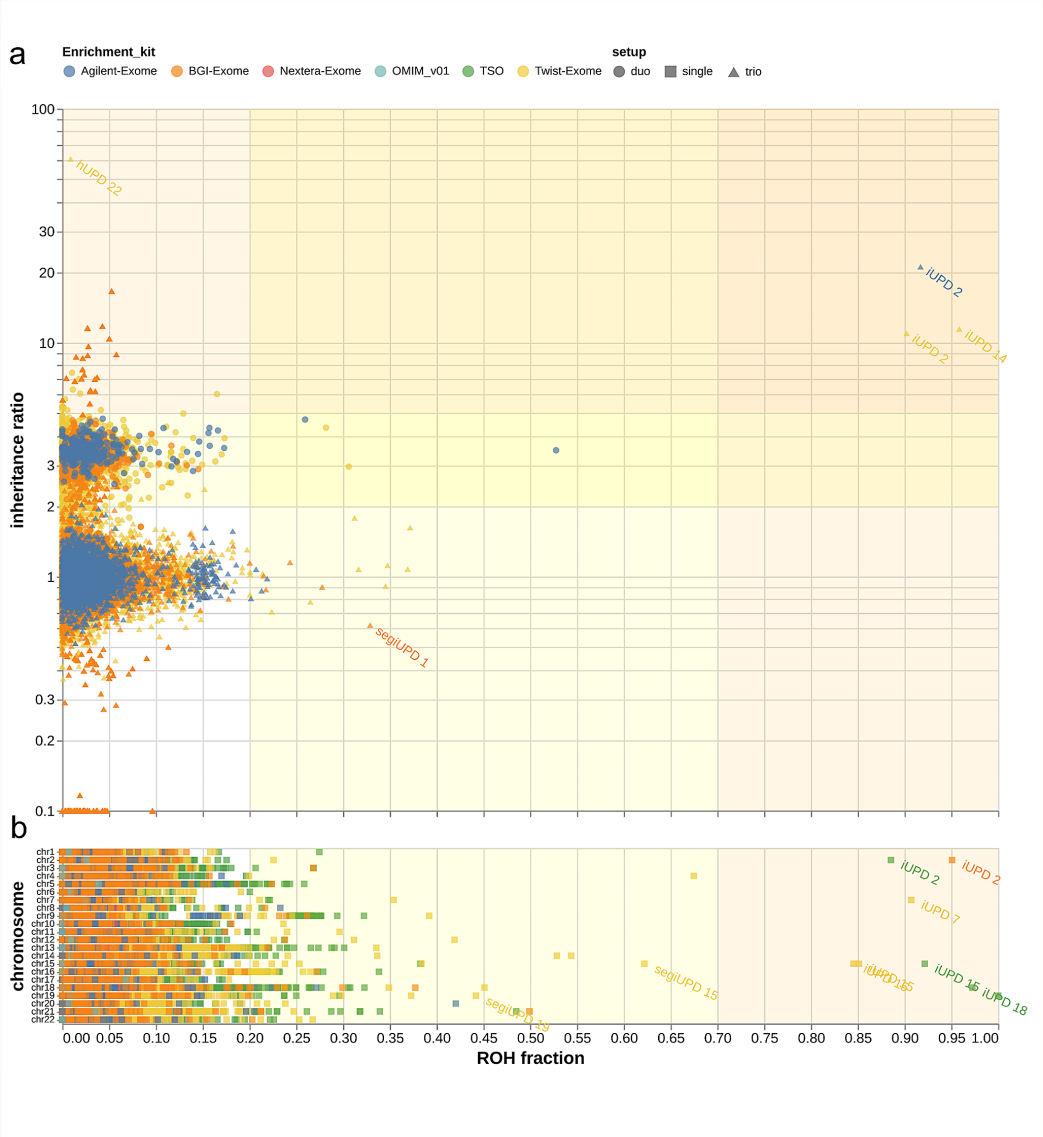
Analysis of NGS data for ROH and IR without consanguineous cases. Symbols indicate different enrichment kits (color, see for details material and methods) and family set-up (shape). Color gradient of the background indicates thresholds for UPD detection: orange area indicates *high probability* for UPD; yellow area indicates *probability* for UPD; white area *indicates low probability* for UPD. Identified UPDs are labeled accordingly **(a)** Trio and duo cohort analysis, each datapoint represents a chromosome of a sample; X-axis represents ROH fraction; y-axis represents inheritance ratio per chromosome. **(b)** Single cohort analysis. X-axis represents ROH fraction; y-axis represents single chromosomes. Abbreviations: hUPD; heterodisomy, iUPD; isodisomy, ROH; region of homozygosity, TSO; TruSightOne Panel, UPD; uniparental disomy.

**Fig. 4:**
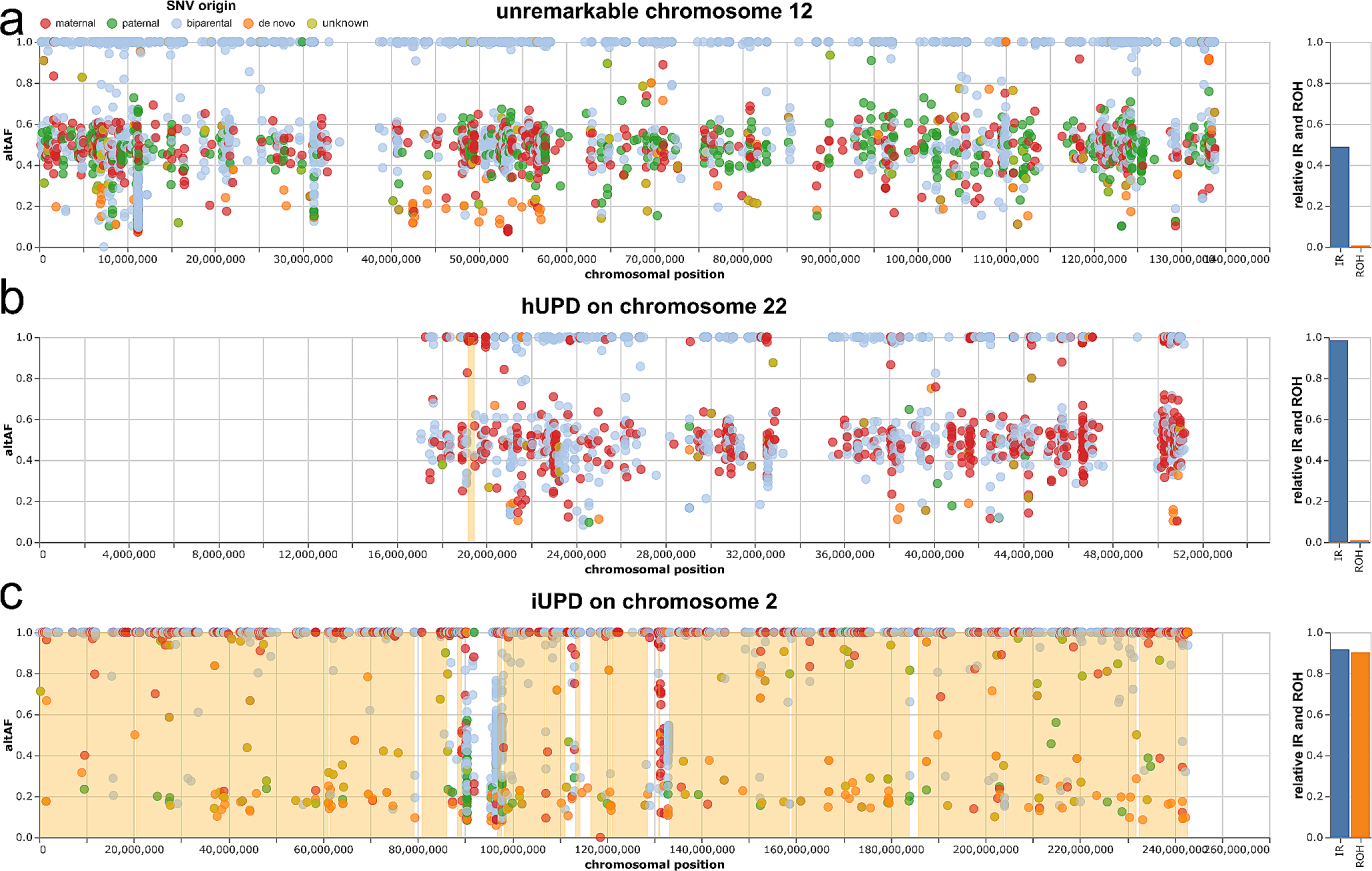
Examples of allele fraction plots. (adapted from the web app altAF-plotter (Radtke et al. 2023)**)**. Colored dots indicate parental origin of the variants, see top left legend. The right panels show relative IRs and ROHs (IR as the fraction of maternal over maternal + paternal variants; ROH as a fraction of the chromosome that is covered by ROHs) **(a)** unremarkable chromosome 12 with equal distribution of paternal and maternal variants and close to zero ROH coverage. **(b)** hUPD on chromosome 22 (P14) showing an increased IR with dominance of maternal variants; paternal variants are missing; ROH coverage is similar as in A **(c)** iUPD on chromosome 2 (P2). Note that the ROH is covering most of the chromosome; IR do not show a clear increase in parental variants (right panel). The variants that remain at an allele frequency of around 0.2 are likely to be artefacts, which are present on every chromosome. In the case of isodisomies all “real” variants have their allele frequency shifted to 1 whereas artefacts remain at a lower frequency and appear as this accumulation of variants at 0.2 AF.

We identified 14 (0.16%) positive UPD findings out of 8556 NGS cases (excluding consanguine cases) comprising ten whole-chromosome UPDs, three segmental UPDs and one heterodisomy (Table 1; Fig. 3). Out of 1557 trio exome analyses we found eight UPD cases (0.5%) including seven isodisomies as well as one heterodisomy on chromosome 22 (Table 1; Fig. 4b, c). Out of our 6853 single exome cases we detected six iUPD cases (0.01%) on different chromosomes (Table 1). Six out of 14 identified UPDs were previously reported in-house but were unknown to us during the analysis. This makes eight findings completely novel. Using IR, we were able to provide information about the inheritance pattern of the identified UPDs in the trio approach. Six UPDs were maternally inherited and five were of paternal origin. This shows an approximately equal inheritance rate of paternal and maternal origin within our cohort.

**Table 1:** Identified UPDs in our cohort. Asterisks indicate newly identified UPDs. Other individuals have been found again. Note that two individuals (P5, P7) had to be excluded from the description as the agreement to data disclosure is missing. Abbreviation: AS; Angelman syndrome, iUPD; isodisomy, hUPD; heterodisomy, segUPD; segmental uniparental disomy, TS; Temple syndrome

Individual	Sex	Phenotype	UPD	Chromosome	Clinical Significance
P1*	male	seizure neurodevelopmental abnormality	segUPD	1	unknown
P2	female	seizure	iUPD	2	unknown
P3	female	seizure neurodevelopmental abnormality	iUPD	2	homozygous variant in *RPIA*
P4	female	seizure neurodevelopmental abnormality	iUPD	2	homozygous variant in *UNC80*
P6	female	growth delay neurodevelopmental abnormality	iUPD	14	TS
P8*	female	seizure neurodevelopmental abnormality	iUPD	15	AS
P9*	male	seizure neurodevelopmental abnormality	iUPD	15	AS
P10	male	seizure neurodevelopmental abnormality	iUPD	15	AS
P11*	male	seizure neurodevelopmental abnormality	segUPD	15	unknown
P12*	female	abnormal muscle tone neurodevelopmental abnormality	iUPD	18	homozygous variant in *PIEZO2* (Moch et al. 2023)
P13*	male	abnormal muscle tone	segUPD	19	unknown
P14*	male	growth delay neurodevelopmental abnormality	hUPD	22	unknown

Out of the 14 UPD events nine were possible causative for the individual’s phenotype through different mechanisms, and underwent clinical reevaluation of their existing ES analysis. Three of these individuals (P3, P4 and P12) carried a homozygous pathogenic or likely pathogenic variant in an autosomal recessive gene inherited from an unaffected parent. The presence of UPDs in the individuals changes the risk of reoccurrence for another pregnancy dramatically in comparison to that of an autosomal recessive inheritance pattern (1% and 25%, respectively) (Erger et al. 2018). Furthermore, six individuals had an imprinting disorder, including four cases of Angelman (P7, P8, P9, P10), one of Temple (P6) and one of Silver-Russell-syndrome (P5). In five cases, the chromosomes affected by UPD are not currently known to be associated with imprinting disorders (Benn 2021). No pathogenic autosomal recessive variants or other chromosomal disturbance could be identified in these cases.

In general, it is difficult to distinguish whether ROHs are caused by UPD or consanguinity in related families (Gonzales et al. 2022) (Supplementary S2). Therefore, we excluded cases in which ROH could not be used as a parameter. For example, cases with deletions known from previous reports, consanguineous families or single cases like Sephardic Jews, a community in which intracommunity marriages can be practiced and can lead to consanguinity over generations (Cohen et al. 2004).

## Discussion

In this study we re-evaluated a cohort of 9212 NGS datasets including exome and multigene panel analyses with our UPD detection pipeline in combination with the web app altAFplotter, based on ROHs and IRs (Radtke et al. 2023). In total, we identified eight UPDs out of 1557 trio datasets and six UPD cases out of 6853 single datasets. In this study we demonstrated the ability to detect isodisomies, heterodisomies as well as segmental UPDs within NGS data independent of the enrichment kit. Approximately half of the identified UPD cases led to the clinical presentation of symptoms due to homozygous recessive variants or as a result of imprinting disorders.

The frequency of UPD in our cohort with approximately 0.16% is relatively high compared to other studies which showed a UPD incidence of 0.05% in a study population consisting of samples from the general population (Nakka et al. 2019) to 0.3% in a study consising of mainly neurological pediatric cases (Scuffins et al. 2021). Reasons for this could be differences in cohort composition between the studies since it has previously been described that the number of pathogenic copy number variations and UPDs is increased in cohorts with more syndromic phenotypes and top-level HPO terms (Dharmadhikari et al. 2019). Our cohort comprised mainly pediatric individuals with several different phenotypic features especially seizures and neurodevelopmental delay which can lead to an accumulation of UPDs in our cohort in comparison to the general population. Furthermore, the number of available parent-child- trio data varies in different studies and the UPD detection in our cohort is higher in trios (0.5%) in comparison to our single cases (0.01%). However, we have also identified all iUPDs from the parent-childset-up in the single analysis which makes the overall frequency of iUPDs in our cohort 0.18%.

Overall, iUPD(15) is the most often described UPD (Liehr 2023). This is probably due to the well-known imprinting disorders Prader-Willi and Angelman syndrome that can occur in context of iUPD(15) (Fridman and Koiffmann 2000). Remarkably, all of our five characterized cases of iUPD(15) are cases associated with Angelman syndrome. This seems to be unusual considering that 25 to 30% of Prader-Willi- and only 2 to 7% of Agelman cases are due to maternal o paternal iUPD(15), respectively (Veltman et al. 2005). However, individuals with Prader-Willi syndrome have a characteristic phenotypic spectrum, including specific facial features (Veltman et al. 2005), which is more readily recognized by clinicians than Angelman syndrome. Thus, the use of targeted methods such as MLPA rather than NGS is likely more often recommended as a first diagnostic step for Prader-Willi cases.

It is also remarkable that we found three cases of iUPD(2)mat within our cohort. The individuals presented with seizures, microcephaly as well as neurodevelopmental delay. Interestingly, two of them have a homozygous variant either in *RPIA* (P3) or *UNC80* (P4) which could explain the individual’s phenotype. So far, the effect of iUPD(2) on an individual’s phenotype in the context of an imprinting disorder is not known and is still being discussed (Gaudio et al. 2020). Interestingly, with 113 reported cases, iUPD(2) does not occur very frequently (Liehr 2023). Still, our finding of three iUPD(2)s is probably no more than a coincidence. Whole methylome sequencing may be useful in the future to investigate the imprinting effects of such unknown UPDs.

Two-thirds of published UPD cases are maternally inherited, which tends to be more frequent in comparison to paternal inheritance (Liehr 2022). Interestingly, this effect is not visible in our cohort since the number of cases with paternal and maternal origin is equal. This could be explained by the high number of Angelman cases within our cohort which elevates the number of paternal cases (Veltman et al. 2005). Likewise, the number of maternal UPDs is linked to advanced maternal age and maternal UPDs are more often heterodisomies in comparison to paternal UPDs which are more often isodisomies (Liehr 2022). This is consistent with our finding that the heterodisomy in our cohort is of maternal origin and all of the paternal inherited UPDs are isodisomies. Furthermore, Scuffins et al. described more heterodisomies affecting smaller chromosomes (Scuffins et al. 2021). This matches the finding of the heterodisomy on chromosome 22. It should be noted, however, that the number of the identified UPDs in this study is not high enough to make a reliable statement on these two topics, especially the occurrence of heterodisomies.

Interestingly, other studies dealing with UPD detection reported that one-third of their cases were heterodisomies (Hoppman et al. 2018). This is surprising considering that only one heterodisomy was present in our cohort of 1557 trio analyses. By validating altAFplotter using positive controls, all included heterodisomies were identifiable using our approach (Yauy et al. 2020; Radtke et al. 2023). This leads to the assumption that our sample size was not large enough to detect heterodisomies at similar frequencies to previous studies. Also including our seemingly not large enough number of parent-child trios which are needed to detect heterodisomies because it is necessary to trace back the parental origin of variants. Increasing the number of parent-child trios in routine diagnostics would be a great benefit for heterodisomy detection.

Furthermore, the detection of UPDs in consanguineous families is challenging as mentioned above. Using ROHs it is hard to differentiate between those caused by iUPD or caused by consanguinity(Gonzales et al. 2022) as well as through duplications or deletions. Including those cases could lead to high numbers of false positive iUPD cases which led to the exclusion of them in our study. An investigation at the exact location of the ROH on the chromosome could be a next step to check if we have to vary our cut-offs depending on the location - intestinal, centromeric or terminal. Since it has been described before, that terminal ROHs are unlikely if UPD or consanguinity are not present (Hoppman et al. 2018). Being alert to terminal ROHs could therefore improve the diagnostic yield of isodisomies even in a single approach.

The investigation of pathogenic effects in context of imprinting disorders related with chromosomes on which no imprinting disorders are known so far, like chromosome 2, using whole-methylome analysis is neccessary. We already started those analysis but the results are not available by now besides those of an iUPD(18)pat case on which we could already successfully detect an altered methylation pattern and described this case separately (Moch et al. 2023). We therefore believe that it is essential to include methylome analysis into future UPD diagnostic processes to advance knowledge about imprinting disorders.

In summary, knowledge of the mechanisms underlying UPD formation is important for the correct assessment of cases with imprinting disorders or homozygous recessive variants, and may have implications for genetic counseling regarding reoccurrence risks. ROHs display a good strategy to detect events of isodisomies, whereas heterodisomy detection requires parent-child duos or trios. We demonstrated that the batchUPDetection workflow in combination with the altAFplotter is a reliable toolset for UPD detection in NGS data and highlighted the identification of heterodisomies with at least one-parent-child analysis. We would also like to point out that our tool works with genomic data as well, since it uses .vcf files. In conclusion, the detection of UPDs in NGS data offers a significant opportunity to increase UPD diagnostic yield to enhance our understanding of imprinting disorders.

### Electronic supplementary material

Below is the link to the electronic supplementary material.

Supplementary Material 1: Figure S1 Phenotypic spectrum of our large cohort including the five most important features of UPDs. Figure S2 Overview of large cohort analysis including probably consanguineous cases

Supplementary Material 2: Table S1 Whole description of the identified UPDs in our cohort.

## Data Availability

No datasets were generated or analysed during the current study.
