# Supplementary material for: Automatized detection of uniparental disomies in a large cohort
Source: Hum Genet. 2024 Jul 16;143(8):955–64. doi: 10.1007/s00439-024-02687-w (PMC11303498; doi:10.1007/s00439-024-02687-w)
Supplement: Supplementary file 1 — Supplementary Material 1: Figure S1 Phenotypic spectrum of our large cohort including the five most important features of UPDs. Figure S2 Overview of large cohort analysis including probably consanguineous cases [file 439_2024_2687_MOESM1_ESM.docx]

**Supplementary Material**


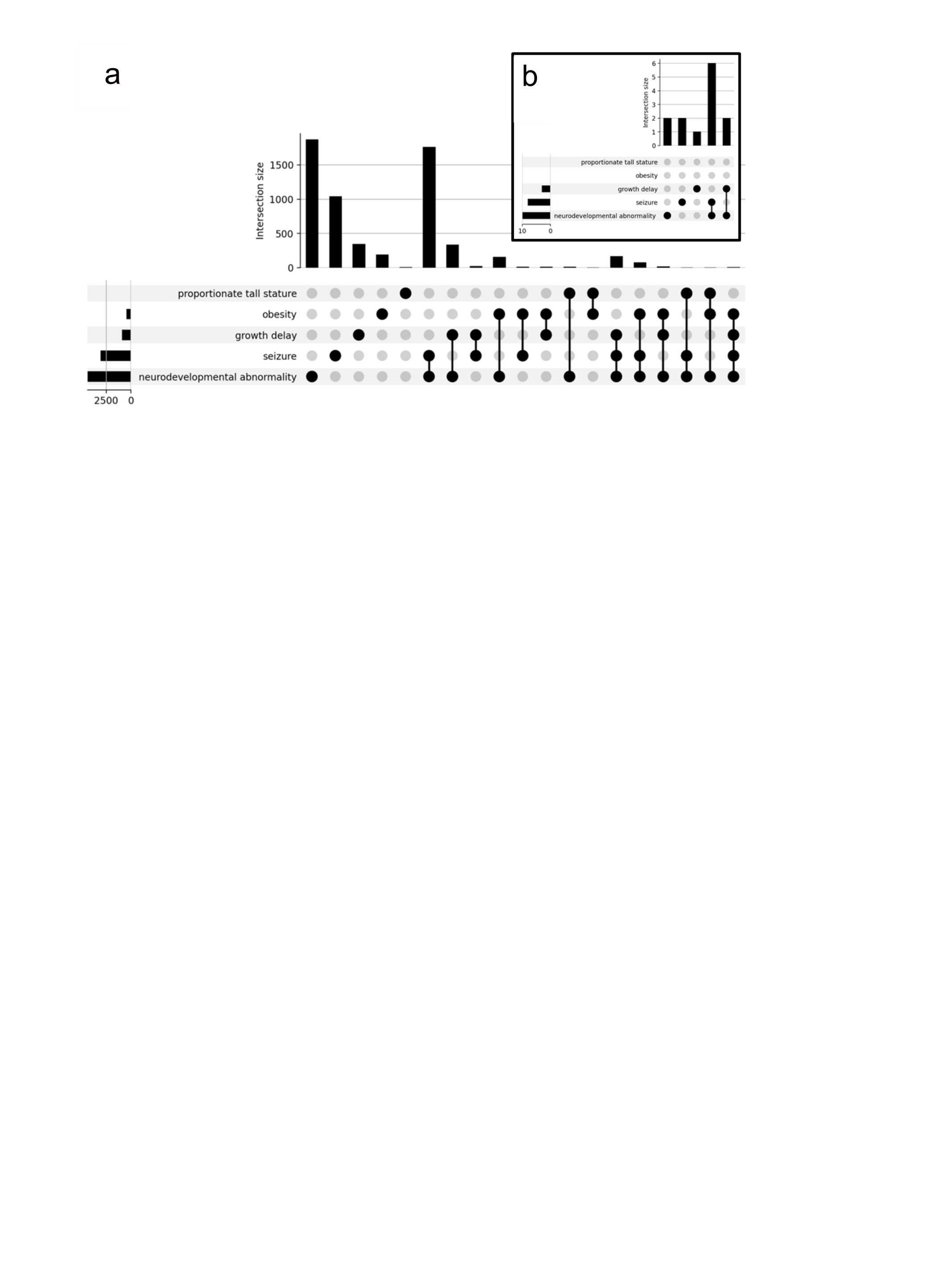


**Figure S1** **Phenotypic spectrum of our large cohort including the five most important features for UPDs.** Each row represents a phenotypic set; each bar charts corresponds to the cohort size for the specific phenotypic set (a) Intersections of our large cohort and five phenotypes. Note that most of the individuals shows neurodevelopmental abnormalities and seizures. (b) Intersections of individuals with an UPD and the five selected phenotypes. Note that tall stature as well as obesity are not represented in the UPD cohort.


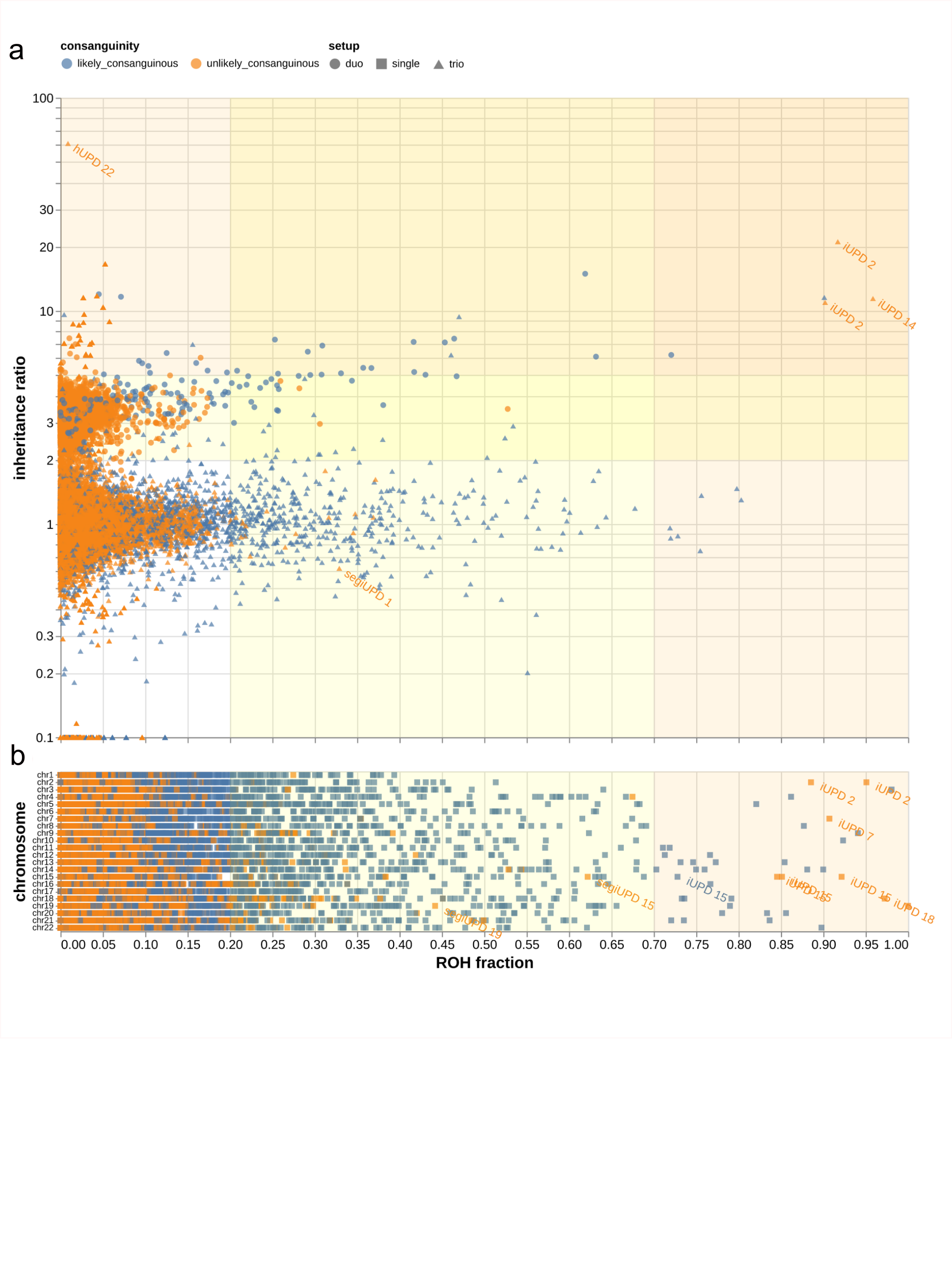


**Figure S2 Analysis of NGS data for ROH and IR with consanguineous cases**. Symbols indicate different enrichment kits (color, see for details material and methods) and family set up (shape). Color gradient of the background indicates thresholds for UPD detection: orange area indicates *high probability* for UPD; yellow area indicates *probability* for UPD; white area *indicates low probability* for UPD. Identified UPDs are labeled accordingly (A) Trio and duo cohort analysis, each datapoint represents a chromosome of a sample; X-axis represents ROH fraction; y-axis represents inheritance ratio per chromosome. (B) Single cohort analysis. X-axis represents ROH fraction; y-axis represents single chromosomes. Abbreviations: hUPD; heterodisomy, iUPD; isodisomy, ROH; region of homozygosity, TSO; TruSightOne Panel, UPD; uniparental disomy.
